# Principles and management of neuropsychiatric symptoms in Alzheimer’s dementia

**DOI:** 10.1186/s13195-015-0096-3

**Published:** 2015-01-29

**Authors:** Milap A Nowrangi, Constantine G Lyketsos, Paul B Rosenberg

**Affiliations:** Division of Geriatric Psychiatry and Neuropsychiatry, Department of Psychiatry and Behavioral Sciences, Johns Hopkins University School of Medicine, 5300 Alpha Commons Dr, 4th Floor, Baltimore, MD 21225 USA

## Abstract

Neuropsychiatric symptoms of Alzheimer’s disease (NPS-AD) are highly prevalent and lead to poor medical and functional outcomes. In spite of the burdensome nature of NPS-AD, we are continuing to refine the nosology and only beginning to understand the underlying pathophysiology. Cluster analyses have frequently identified three to five subsyndromes of NPS-AD: behavioral dysfunction (for example, agitation/aggressiveness), psychosis (for example, delusions and hallucinations), and mood disturbance (for example, depression or apathy). Recent neurobiological studies have used new neuroimaging techniques to elucidate behaviorally relevant circuits and networks associated with these subsyndromes. Several fronto-subcortical circuits, cortico-cortical networks, and neurotransmitter systems have been proposed as regions and mechanisms underlying NPS-AD. Common to most of these subsyndromes is the broad overlap of regions associated with the salience network (anterior cingulate and insula), mood regulation (amygdala), and motivated behavior (frontal cortex). Treatment strategies for dysregulated mood syndromes (depression and apathy) have primarily targeted serotonergic mechanisms with antidepressants or dopaminergic mechanisms with psychostimulants. Psychotic symptoms have largely been targeted with anti-psychotic medications despite controversial risk/benefit tradeoffs. Management of behavioral dyscontrol, including agitation and aggression in AD, has encompassed a wide range of psychoactive medications as well as non-pharmacological approaches. Developing rational therapeutic approaches for NPS-AD will require a firmer understanding of the underlying etiology in order to improve nosology as well as provide the empirical evidence necessary to overcome regulatory and funding challenges to further study these debilitating symptoms.

## Introduction

Alzheimer’s disease (AD) is the most common neurodegenerative condition in aging. AD is a growing public health problem that is projected to reach epidemic proportions if disease-modifying therapies are not found. The latest figures from the Alzheimer’s Association indicate that an estimated 5.3 million Americans live with AD. By 2050, an estimated 11 to 16 million people are expected to be diagnosed in the US alone [1]. Data from Alzheimer’s Disease International forecast that the worldwide prevalence of AD will double every 20 years to 65.7 million by 2030 and 115.4 million by 2050 with higher proportions in the developed versus undeveloped countries [2-4]. The economic costs of caring for persons with AD are staggering. The Alzheimer’s Association estimates that total health-care costs for persons with AD are expected to top $172 billion, including $123 billion in costs to Medicaid and Medicare alone [5], even without taking into account the ‘indirect costs’ of providing care such as caregiver loss of productivity and use of long-term care facilities and hospice.

The dementia syndrome stemming from the pathological processes underlying AD results in cognitive, functional, and behavioral symptoms [6]. Diagnosis of dementia due to AD is based primarily on the clinical presence of cognitive and functional impairment. The most recent consensus statement from the National Institute on Aging and the Alzheimer’s Association workgroup has proposed revised criteria incorporating biomarker evidence of probable AD and represents a step forward in the clinical evaluation of AD [7-9]. Although there has been progress in developing diagnostic modalities, there has been less progress in the management of the pathognomic cognitive symptoms and functional sequelae and even less for the behavioral management of AD.

Neuropsychiatric symptoms of AD (NPS-AD) are multidimensional behavioral phenotypes commonly found in AD. NPS-AD are highly prevalent (80% to 90%), and most individuals with AD will exhibit one or multiple symptoms over the course of the illness [10-12]. Symptoms can range from mild (depression, anxiety, irritability, and apathy) to severe (agitation, aggression, aberrant vocalizations, hallucinations, and disinhibition, among others). These symptoms have been shown to persist or recur over time and are associated with patient and caregiver distress, increased rates of institutionalization, and increased mortality [13-16]. Management of these symptoms has relied on the use of both pharmacological and non-pharmacological therapies based on treatments developed for idiopathic psychiatric disorders. A key reason for the limited effectiveness and lack of progress for the development of these treatments is an incomplete understanding of the biological mechanisms responsible for these symptoms. As such, current treatment strategies are considered symptomatically driven and treatment development largely empiric rather than being based on a mechanistic understanding. Thus, we are lacking an essential piece in rational therapeutics.

In an effort to better understand the management of NPS-AD, this article will review the current literature on the phenomenology and diagnosis, neurobiology, and treatment approaches for several key behavioral phenotypes in AD. It is hoped that a summary of these findings at this point will encourage further research by demonstrating gaps in our knowledge of these conditions while emphasizing the growing burden of these symptoms on patients and caregivers as well as the need for more effective therapies.

## Phenomenology, detection, and classification of neuropsychiatric symptoms of Alzheimer’s disease

Understanding the presentation of NPS-AD has generally followed inductive reasoning. Colloquially known as the ‘duck test’ approach, similarities between two ideas or objects or, in this case, conditions are made and conflated or used interchangeably. That is, ‘if it walks like a duck, swims like a duck, quacks like a duck, it must be a duck’. These are essentially analogs of symptoms from idiopathic psychiatric disorders (that is, disorders of emotion, thought, or behavior for which there is no definitive pathophysiology). For example, if an individual with AD were to exhibit lack of reactivity or report low mood, poor appetite, or disordered sleep, a diagnosis of depression in AD may be given. Similarly, if an individual exhibits hallucinations or delusions or psychomotor activation, a diagnosis of psychosis may be offered to explain the phenomenon. Generally then, the descriptions of these phenomena, based on first-person experience (direct and primary observation), generate diagnoses, which direct treatment decisions.

Detection of NPS-AD has relied primarily on caregiver-reported scales. The most commonly used scale is the Neuropsychiatric Inventory (NPI) [17,18], which has been considered the standard assessment for neuropsychiatric symptoms for the past two decades by assessing symptoms in 12 domains (Table 1). Recently, a revised version of the NPI, the Neuropsychiatric Inventory Clinician reported (NPI-C) [19], has been developed to address several shortcomings of the NPI and other rating scales and is a well-validated and commonly used scale. A major improvement of the NPI-C was the addition of a clinician rating methodology, which mitigates the reliance on caregiver-provided information and in so doing systematizes the way in which clinical observation and caregiver report are integrated. There are alternate versions of the NPI, including (a) NPI-NH [20], designed for use in the nursing home setting, and (b) the NPI-Q [21], a brief caregiver report questionnaire that can be completed in 5 minutes or less and has good test-retest reliability and convergent validity correlating with the full NPI at 0.91 [21,22].

**Table 1:** **Neuropsychiatric Inventory questionnaire domains**

Delusions	ᅟ
Hallucinations	
Agitation or aggression	
Depression or dysphoria	
Anxiety	
Elation or euphoria	
Apathy or indifference	
Disinhibition	
Irritability or lability	
Motor disturbance	
Night-time behavior	
Appetite and eating	

As a result of these empirical approaches, the search for etiology has generated and continues to generate biological and neuropathological as well as psychosocial hypotheses. This has led to considerable heterogeneity in classifying these symptoms. Subcategorizing by associative clustering has been one approach to understanding potentially differential prevalences, trajectories, and biological mechanisms. One of the earliest attempts of this was with the Cache County study, in which 198 individuals with AD were identified in a cohort of 5,092 older residents [23]. When the NPI and neuropsychiatric evaluation were used, three latent classes of symptoms were identified: primarily affective symptoms (28%), primarily psychotic symptoms (13%), and mono-symptomatic disturbance (19%); the remainder had no symptoms (40%). More recent cross-sectional and longitudinal studies have gone on to similarly describe three to five factors by using principal component factor analysis [24]. A study by Vilalta-Franch and colleagues [25] further described a ‘hypomanic’ group in which euphoria and disinhibition clustered together.

The elucidation of these factors has been the result of numerous studies frequently employing latent class analysis, in which the latent factor is expressed as the mathematical property of all the observed variables [26-31]. Because of this and other statistical challenges inherent in exploratory factor analysis employed by earlier studies, Cheng and colleagues [32] recently used an alternative method in which the statistical approach started by using hypothetical behavioral syndromes that made ‘clinical sense’ to guide confirmatory factor analysis. Their analysis supported a four-factor model: (1) behavioral problems (agitation/aggressiveness, disinhibition, irritability, and aberrant motor behavior), (2) psychosis (delusions and hallucinations), (3) mood disturbance (depression, sleep, appetite, and apathy), and (4) euphoria. These studies appear to provide an evidence-based foundation for the classification of NPS-AD and further represent an evolution in the nosology of these symptoms; however, the lack of consistency and presence of contradictions within the available data continue to be limitations. A systematic review by Canevelli and colleagues [24], who evaluated the evidence coming from factor analyses involving the use of the NPI, showed a relatively low concordance among available evidence for clusters represented in the NPI. In fact, no study reported the same cluster composition of another one. However, this review did find consistent association of specific symptoms across studies that may potentially define behavioral subsyndromes of AD: delusions and hallucinations, depression and anxiety, agitation and irritability, and euphoria and disinhibition were found to be frequently associated symptoms characterizing possible phenotypes as previously mentioned. Nonetheless, there remain considerable gaps in our knowledge primarily driven by our nascent understanding of the underlying biology.

## Neurobiology of neuropsychiatric symptoms of Alzheimer’s disease

Although there have been significant advances in understanding the functional neuroanatomy, neurochemistry, and neurophysiology of cognitive phenotypes (memory, language, visuospatial ability, and executive functions), there has been less progress in understanding more complex behavioral phenotypes associated with AD. State-of-the-art structural and functional neuroimaging approaches have been widely used to study the core pathological processes of AD and are just starting to be applied to NPS-AD. For these reasons, current research in the neurobiology of NPS-AD is driven by accepting the assumptions first that neuropsychiatric symptoms are accurately measured in AD and second that NPS-AD is related to changes in brain structure and function that point toward underlying mechanisms of behavior that may or may not be shared with other neuropsychiatric or idiopathic psychiatric disease. The implication of brain circuits and networks as revealed through current research reflected a paradigm shift in understanding the relationship between brain and behavior [33]. Born out of Norman Geschwind’s pioneering work in disconnection syndromes, a ‘disconnectionist’ framework provided the field with the understanding that higher function deficits resulted from lesions of white matter in association cortices that act as relay stations between primary motor, sensory, and limbic areas [34]. With the advent of neuroimaging and other neurophysiological techniques, it has now become possible to study connections *in vivo* and make correlations between disconnecting lesions or processes and clinical symptoms.

As part of a recent initiative by the Alzheimer’s Association, the Neuropsychiatric Syndromes of AD Professional Interest Group of the International Society to Advance Alzheimer’s Research and Treatment, Geda and colleagues [35] recently put forth a tripartite hypothetical model based on empirical research to guide the further study of NPS-AD. The first hypothesis is that at least three frontal-subcortical circuits (dorsolateral prefrontal circuit, orbitofrontal prefrontal circuit, and circuit involved in motivated behavior) mediate human behavior. The second hypothetical model posits that cortico-cortical networks, of which five have been frequently cited, mediate emotional as well as cognitive processing. Finally, the third model suggests that the monoaminergic system involving such neurotransmitters as serotonin, norepinephrine, and dopamine along white matter projections from rostral to caudal brain regions mediate complex behavior. Certainly, modulation of glutamatergic [36], neurotrophic (for example, brain-derived neurotrophic factor) [37], and other neurochemical and neuroendocrine systems has also received attention as related mechanisms of action [38]. A brief review of specific contributions of neurotransmitter and neuroimaging studies to our understanding of mechanisms of NPS-AD are summarized in Table 2 and are expanded upon below.

**Table 2:** **Summary of neurobiological correlates of neuropsychiatric symptoms of Alzheimer’s disease and their treatments**

**Neuropsychiatric symptoms of Alzheimer’s disease**	**Neurotransmitter mechanisms**	**Neuroimaging correlates**	**Treatments**
Depression	Monoaminergic, noradrenergic, gamma amino butyric acid (GABA) neurotransmission dysfunction	Reduced entorhinal cortex thickness; accelerated atrophy in anterior cingulum; decreased cerebral glucose in frontal and parietal cortex	Serotonin (serotonin selective reuptake inhibitor, or SSRI), norepinephrine (serotonin-norepinephrine reuptake inhibitor, or SNRI), non-pharmacological treatments
Apathy	Lower dopamine transporter binding; lower cholinergic receptor binding	Decreased metabolic activity in anterior cingulate and orbitofrontal cortex; functional deficits in several medial and inferior frontal regions	Methylphenidate, amantadine, d-amphetamine, modafanil, non-pharmacological treatments
Agitation and aggression	Cholinergic neurotransmission deficits; increased D2/D3 receptor availability; monoaminergic (5-HT2A) transmission defects	Cortical atrophy of cingulum and frontal gyrus; insula, amygdala, and hippocampal atrophy; lower metabolic activity in temporal frontal and cingulum; anterior salience network	Citalopram, atypical anti-psychotics, anti-epileptic mood stabilizers, non-pharmacological treatments
Psychosis	D2/D3 receptor availability, monoaminergic, cholinergic	Lower regional cerebral blood flow in angular gyrus and occipital lobe; increased atrophy in neocortical, frontal, parietal and cingulum	Atypical anti-psychotics, non-pharmacological treatments

### Depression

There is evidence for alterations in monoaminergic neurotransmitter functioning and brain metabolism underlying depression in AD. For example, there is evidence for selective loss of 5HT1A receptors in the hippocampus [39] as well as loss of noradrenergic neurons in locus coeruleus and serotoninergic neurons in the raphe nucleus [40-42]. These findings represent additional functional loss of serotonergic neurotransmission over and above the substantial losses observed in AD in general [43,44] which selectively affect 5HT2A receptors in early AD [45]. However, basal forebrain cholinergic neurons are relatively spared in depressed patients with AD [46,47]. Additionally, there is evidence for decreased gamma amino butyric acid (GABA) levels and greater number of GABA (A) receptors in depressed patients with AD [48]. Studies of genetic polymorphisms in serotonin and dopaminergic transporters and receptors have not distinguished depressed versus non-depressed patients with AD. Zahodne and colleagues [49] reported that depression in mild cognitive impairment (MCI) was associated with reduced cortical thickness in the entorhinal cortex at baseline and accelerated atrophy in the anterior cingulate cortex. They interpreted this to mean that depression was associated with the overall rate of neurodegeneration in AD or with reduced cognitive reserve. Several studies have demonstrated lower cerebral glucose metabolism in depressed versus non-depressed patients with AD in the frontal (dorsolateral prefrontal cortex, superior frontal gyrus, and anterior cingulate gyrus) or parietal cortex (post-central gyrus and superior and inferior lobule) [50,51]. One study observed that lower 5-HTT transporter density was correlated with greater depression symptom severity [52]. These findings are in contrast to late-life major depression, including the observations of greater white matter hyperintensities, increased cortical glucose metabolism, and 5-HTT and 5-HT1A receptor loss, reviewed in Khandai and Aizenstein [53] (2013) and Hirao and Smith [54] (2014). Thus, the evidence indicates that decreased monoaminergic neurotransmitter function and decreased fronto-parietal metabolism are involved in mechanisms of depression in AD and that the metabolic changes are opposite to those observed in late-life major depression.

### Apathy

Lower dopamine transporter binding has been associated with decreased initiative [55], whereas lower cholinergic receptor binding in the left frontal cortex has been reported to be associated with motor and mood changes of apathy [56]. These findings mirror successful treatment of apathy in AD with the dopaminergic agent methylphenidate (see below). Apathy has been associated with decreased metabolic activity in anterior cingulate and orbitofrontal cortex. There is growing evidence that apathy in AD is associated with structural atrophy and functional deficits in several medial and inferior frontal regions thought to mediate motivation and reward mechanisms [35]. Starkstein and colleagues [57] reported that apathy was associated with greater volume of white matter hyperintensities in frontal cortex, suggesting a greater burden of small-vessel ischemic disease. Marshall and colleagues [58] reported that apathy in AD was associated with decreased metabolic activity in anterior cingulated and orbitofrontal cortex.

### Agitation and aggression

There is evidence that agitation/aggression in AD is related to dysfunction of specific brain regions. There is growing evidence that structural and functional deficits may be related to deficits in cholinergic neurotransmission (over and above those seen in AD itself) and with increased D2/D3 receptor availability in the striatum. An important study by Assal and colleagues [59] showed a positive association between 5-HT2A receptor gene polymorphism and agitation and aggression in AD, suggesting a role for serotonin transmission defects. Serra and colleagues [60] reported that disinhibition was strongly associated with grey matter atrophy in bilateral anterior cingulate and right middle frontal gyri. Bruen and colleagues [61] reported a similar association of agitation with grey matter atrophy in bilateral anterior cingulate and additionally in left insula. In a larger cohort of participants in the Alzheimer’s Disease Neuroimaging Initiative, Trzepacz and colleagues [62] reported an association of agitation severity with greater atrophy of frontal, insular, amygdala, cingulate, and hippocampal regions. Reeves and colleagues [63] reported that striatal dopamine receptor D2/D3 binding - as measured by 11C-raclopride positron emission tomography (PET) - was associated with severity of delusions and disinhibition in AD. Sultzer and colleagues [64,65] reported that agitation in AD correlated with lower metabolic activity (as measured by fluorodeoxyglucose-PET) in right lateral temporal, right lateral frontal, and bilateral cingulate cortex. Mielke and colleagues [66] reported that lower structural integrity of the fornix (as measured with diffusion tensor imaging) was associated with agitation in AD. Balthazar and colleagues [67] reported that the NPI ‘hyperactivity factor’ (including agitation) was associated with greater resting-state functional connectivity in the anterior salience network, particularly in right anterior cingulate and insula. When the biological evidence is fully considered, it appears that agitation/aggression in AD is associated with cortical dysfunction in anterior cingulate, insula, lateral frontal, and lateral temporal regions, many of which are associated with the anterior salience network thought to direct brain resources toward specific cognitive activities [68].

### Psychosis

A number of neurotransmitter systems have been implicated in psychosis of AD. Increased availability of striatal dopamine (D2/D3) as well as increased dopamine D3 receptor density in the nucleus acumbens has been found in AD patients with psychosis, compared with those without psychosis [69]. Additionally, a lower density of 5-HT receptors has been found in the ventral temporal cortex and prosubiculum as well as increased ratio of acetylcholinesterase/5-HT [70,71]. It has been suggested that the lower 5-HT levels may be associated with lower cell counts in the dorsal raphe nucleus in AD patients with psychosis [72]. With regard to neuroanatomy, a study by Banno and colleagues [73] showed that, when studied with single-photon emission computed tomography imaging, psychosis symptoms in AD were most associated with lower regional cerebral blood flow values in the right angular gyrus (Brodmann area 39) and the right occipital lobe (Brodmann area 19). Rafii and colleagues [74] showed that regional atrophy rates in neocortical, lateral frontal, lateral parietal, and anterior cingulate gyrus were significantly associated with the onset of psychosis, including delusions, agitation, wandering, and hallucinations and/or the need for anti-psychotic medications. Findings from this study in particular are consistent with other studies that similarly identify a well-characterized corticosubcortical circuit involving the frontal gyri and the cingulate gyrus purportedly involved in mediating complex intentional and motivated behaviors as well as high-order cognitive functions such as planning and problem solving [75-77]. It is suggested, then, that a disconnection of this and other neocortical networks is related to the presence of psychosis in AD.

What is most striking about the data from agitation, depression, apathy, and psychosis in AD is the broad overlap of regions associated with each NPS syndrome, particularly regions known to be associated with the salience network (anterior cingulate and insula), mood regulation (amygdala), and motivated behavior (frontal cortex). This suggests at least partial overlap of brain mechanisms subsuming these NPS.

## Management of common neuropsychiatric syndromes in Alzheimer’s disease

The most common NPS-AD are depression, apathy, delusions and hallucinations, and agitation and aggression, with point and 5-year prevalence ranging between 20% and 50% according to the Cache County cohort [14]. These symptoms can be and have been clustered into affective, psychotic, and behavioral dyscontrol syndromes as discussed earlier. Given this high prevalence as well as the deleterious social and financial effects, we now turn our focus to management principles of these syndromes.

### Management of depression and apathy in Alzheimer’s disease

Depression and apathy are the most common affective symptoms in AD. Depressive symptoms have been hypothesized to be related in part to disturbances in the monoaminergic system involving neurotransmitters such as serotonin, norepinephrine, and dopamine in non-AD conditions such as major depression as referenced earlier. As such, the majority of pharmacological interventions are targeted at alleviating depressive symptoms primarily to improve quality of life and improve functioning in AD and secondarily to mitigate factors exacerbating cognitive impairment and hindering the efficacy of pro-cognitive drugs and other non-pharmacological interventions. Anti-depressant medications have been most frequently studied in comorbid depression in AD. A recent meta-analysis by Sepehry and colleagues [78], however, paints a rather unenthusiastic picture for the use of anti-depressant medications. After applying inclusion and exclusion criteria, the authors selected six studies/trials from which an aggregate of 297 participants enrolled in treatment arms and 318 enrolled in placebo arms. Via random-effects modeling, the meta-analysis found non-significant effects in two depression nested analyses using the Cornell Scale for Depression in Dementia and the Hamilton Depression Rating Scale. In spite of this, the authors caution that clinical choice of specific serotonin selective reuptake inhibitors other than sertraline, which the majority of the selected studies tested, may differentially affect outcomes, including potentially positive effects because of differences in pharmacological action on serotonin receptor subunits. Studies testing serotonin-norepinephrine reuptake inhibitor medications did not meet this meta-analysis inclusion criteria, but studies of venlafaxine [79], for example, have resulted in mixed findings.

Apathy is differentiated from depression in that individuals suffer from diminished motivation as demonstrated by decreased goal-directed behavior, decreased goal-directed cognitive activity, and constricted or blunted affective range. Neurobiologically, apathy has been associated with dysfunction of the prefrontal and anterior cingulate regions, including both cortical and subcortical regions, with causes ranging from volume loss to increased burden of white matter hyperintensities to decreases in regional cerebral blood flow to increases in prefrontal Aβ deposition [80]. Moreover, overlaps found between apathy and executive dysfunction have caused some speculation that the thalamic-prefrontal-subcortical circuitry is shared between the two. Unfortunately, few controlled trials target treatment of apathy in AD. One recent randomized placebo-controlled trial of methylphenidate for apathy in AD (ADMET) reported improvement in two out of three efficacy outcomes with a trend toward improved global cognition with minimal adverse events [81-83]. Few other uncontrolled trials, however, have also shown treatment effects with methylphenidate [84,85] and one with donepezil [86]. Other medications, including amantadine [87], d-amphetamine [88], and modafanil [89], have shown mixed results.

### Management of psychotic symptoms in Alzheimer’s disease

Individuals with AD and psychotic symptoms tend to suffer greater functional decline, increased rates of institutionalization, and increased mortality and increased caregiver burden even after controlling for age, gender, cognitive and functional disability, general health status, and use of anti-psychotic treatment [90]. The symptoms are characterized by auditory or visual hallucinations (or both) and delusions such as paranoia and persecution.

Treatment of psychotic symptoms in AD is based on severity and frequency of symptoms as well as the disability resulting from them and weighing risks and benefits of using anti-psychotic medications when other non-pharmacological measures and potentially medical causes have been ruled out. Use of anti-psychotic medications such as atypical and typical neuroleptic medications should be reserved as a last resort because of the potentially dangerous side effects and limited to modest benefit these provide. Large-scale meta-analyses consistently demonstrate a 1.5- to 1.7-fold increase in mortality when atypical anti-psychotic medications are used [91,92]. As a result, the US Food and Drug Administration has included a ‘black-box’ warning for atypical anti-psychotics, cautioning about increased mortality and risk of cerebrovascular accidents. Other adverse effects include cardiovascular and metabolic derangements, extrapyramidal motor symptoms, cognitive worsening, infections, and falls. The large Clinical Antipsychotic Trials of Intervention Effectiveness Alzheimer’s Disease trial showed non-significant treatment effects of three anti-psychotics (olanzapine, quetiapine, and risperidone) when compared with placebo. Moreover, time to discontinuation due to intolerable side effects was higher for those taking the anti-psychotics versus placebo [93,94]. Results from a recent study by Lopez and colleagues [95] challenge these findings and warnings by showing that the subset of their cohort exposed to neuroleptic drugs did not show a relationship to increased mortality or increased institutionalization when controlling for the psychiatric symptoms for which the drugs were prescribed. These results suggest that, when adjusting for relevant covariates, the presence of psychiatric symptoms, including psychosis and agitation, was linked to poor outcomes rather than the medications themselves. Taken together, these results as well as the limited efficacy of these medications point to significant gaps in our knowledge of the specific neurobiology of these symptoms in AD compared with other psychotic disorders such as schizophrenia.

### Management of agitation and aggression in Alzheimer’s disease

Agitation and aggression have frequently been termed ‘dysexecutive’ symptoms because of their relationship to ‘executive’ or higher-order loss of behavioral control [96-98]. Relevant domains on the NPI-C include agitation, aggression, irritability/lability, aberrant motor behavior, and aberrant vocalizations. Disruptions in prefrontal-subcortical-thalamic circuits as well as other cortico-cortical circuits have been proposed as neuroanatomical substrates related to these symptoms as previously discussed. Neurotransmitter systems along these circuits have included dopaminergic, noradrenergic, serotonergic, acetylcholinergic, and other systems as potentially exerting some modulatory effect. In spite of the associated neurobiology, the literature continues to lack general agreement on either symptom classification or identification and treatment. For example, serotonergic treatment with citalopram in the Citalopram for Agitation in AD study [99,100] observed significant improvement on outcome measures of agitation that was clinically meaningful on a global functioning scale compared with those who received placebo, whereas studies of dopamine augmentation [101] with medication such as amantadine have shown some modest treatment effects [102]. Use of atypical anti-psychotic medications (see above) as well as anti-epileptic mood stabilizers such as valproic acid [103-105] has become a mainstay in the treatment of these behaviors but similarly carries considerable risk.

### Non-pharmacological treatments

Non-pharmacological treatments (NPTs) encompass such interventions as cognitive stimulation, cognitive training, behavioral interventions, exercise, music, and multi-sensory stimulation for those with dementia. NPTs can also be guided toward caregivers and include education, support, case management, and respite care. As such, a wide range of different types of therapies have been studied. Some small studies have shown modest benefit with strategies such as aromatherapy, bright light therapy, and music therapy. Behavior therapy using antecedent-behavior-consequence (ABC) diary assessment has provided good sustained improvements in behavior [106,107]. One meta-analysis found that behavioral management techniques focused on individual patients’ behavior and individually oriented psychoeducation provided longer-lasting (several months) positive effects on behavior when compared with placebo [106]. Music therapy and Snoezelen were two other types of therapies that provided positive yet short-lived effects. Finally, staff education has been shown to lead to reductions in behavioral outbursts and fewer episodes of restraint use. Despite the promising results from these studies, there is little controlled evidence that they in fact work, and they are often difficult to implement in real-world settings. In a meta-analysis by Olazarán and colleagues [108], the majority of the studies (randomized controlled trials) reviewed showed positive effects for improvements in mood, behavior, and quality of life of persons with dementia while delaying institutionalization. The quality and depth of these studies should continue to be improved upon to further provide empirical support for the use of NPT in AD. Funding for this type of work, unfortunately, has become a limitation.

## Conclusion: treatment development and where to go from here

Rationally developing disease and symptom-specific therapies such as those for NPS-AD requires a firmer understanding of etiology. In this case, advances are being made to further elucidate the neural underpinnings of NPS-AD and will include contributions from neuroimaging, etiopathological studies, and other areas of basic and clinical neuroscience to test the hypotheses put forth by Geda and colleagues [35]. With increasing focus on pre-morbid states such as MCI, studying the link between NPS in MCI, mild behavioral impairment [109], and dementia onset should also provide significant insight into etiology. Understanding brain-behavior relationships in terms of neural circuits and behaviorally (as well as cognitively) relevant networks has been an important development which has revolutionized the way we think about designing and developing as well as monitoring the effects of interventions. Further research into developing the technologies that will be used to elucidate these substrates ought to become a major area of focus over the next decade. In the interim, continued development of patient/caregiver and clinician assessment tools such as the NPI should continue to be standardized for different populations of individuals.

Important challenges to continued research in this area include regulatory and funding limitations. Definitions of these symptoms continue to garner inconsistency and disagreement and have been a fundamental gap in understanding (and naming) disease syndromes as well as obtaining regulatory approval and governmental funding. Certainly, progress is being made as definitions of depression, apathy, and psychosis have gained increasing levels of consensus. Definitions of agitation and other neuropsychiatric symptoms require further work in describing the phenomena and classifying them. In order for the US Food and Drug Administration to ‘carve out’ additional NPS targets for drug development, well-defined symptom clusters must be provided and be supported by widespread acceptance in the clinical/academic communities. Another challenge would be to posit and defend the hypothesis that NPS-AD such as depression is distinct in terms of phenomenology, course, and response when compared with idiopathic depression, for example. Bolstering efforts toward improving clinical epidemiological studies that validate proposed syndromes, identify risk and protective factors, and provide high-quality naturalistic study designs will be significant steps forward and provide further empirical evidence needed to support applications for funding. With growing pathophysiological knowledge, studies directed at testing efficacy of novel therapies and especially ones that integrate and sequence non-pharmacological and pharmacological approaches will provide a comprehensive approach that sets up discontinuation, safety, and long-term outcome studies.

## Abbreviations

AD: Alzheimer’s disease
GABA: Gamma amino butyric acid
MCI: Mild cognitive impairment
NPI: Neuropsychiatric Inventory
NPI-C: Neuropsychiatric Inventory Clinician reported
NPS-AD: Neuropsychiatric symptoms of Alzheimer’s disease
NPT: Non-pharmacological treatment
PET: Positron emission tomography

## References

[1] Alzheimer’s Association (2014). Alzheimer’s disease facts and figures. Alzheimers Dement.

[2] Prince M, Jackson J (2009). World Alzheimer Report 2009.

[3] Department of Economic and Social Affairs (2007). World Population Aging 2007.

[4] Kalaria RN, Maestre GE, Arizaga R, Friedland RP, Galasko D, Hall K (2008). Alzheimer’s disease and vascular dementia in developing countries: prevalence, management, and risk factors. Lancet Neurol.

[5] Association A’s (2010). Alzheimer’s disease facts and figures. Alzheimers Dement.

[6] Nowrangi MA, Rao V, Lyketsos CG (2011). Epidemiology, assessment, and treatment of dementia. Psychiatr Clin North Am.

[7] Jack CR, Albert MS, Knopman DS, McKhann GM, Sperling RA, Carrillo MC (2011). Introduction to the recommendations from the National Institute on Aging- Alzheimer’s Association workgroups on diagnostic guidelines for Alzheimer’s disease. Alzheimers Dement.

[8] McKhann G, Drachman D, Folstein M, Katzman R, Price D, Stadlan EM (1984). Clinical diagnosis of Alzheimer’s disease: report of the NINCDS-ADRDA Work Group under the auspices of Department of Health and Human Services Task Force on Alzheimer’s Disease. Neurology.

[9] McKhann GM, Knopman DS, Chertkow H, Hyman BT, Jack CR, Kawas CH (2011). The diagnosis of dementia due to Alzheimer’s disease: recommendations from the National Institute on Aging-Alzheimer’s Association workgroups on diagnostic guidelines for Alzheimer’s disease. Alzheimers Dement.

[10] de Vugt ME, Riedijk SR, Aalten P, Tibben A, van Swieten JC, Verhey FR (2006). Impact of behavioural problems on spousal caregivers: a comparison between Alzheimer’s disease and frontotemporal dementia. Dement Geriatr Cogn Disord.

[11] Steinberg M, Tschanz JT, Corcoran C, Steffens DC, Norton MC, Lyketsos CG (2004). The persistence of neuropsychiatric symptoms in dementia: the Cache County Study. Int J Geriatr Psychiatry.

[12] Tariot PN, Mack JL, Patterson MB, Edland SD, Weiner MF, Fillenbaum G (1995). The Behavior Rating Scale for Dementia of the Consortium to Establish a Registry for Alzheimer’s Disease. The Behavioral Pathology Committee of the Consortium to Establish a Registry for Alzheimer’s Disease. Am J Psychiatry.

[13] Shin IS, Carter M, Masterman D, Fairbanks L, Cummings JL (2005). Neuropsychiatric symptoms and quality of life in Alzheimer disease. Am J Geriatr Psychiatry.

[14] Steinberg M, Shao H, Zandi P, Lyketsos CG, Welsh-Bohmer KA, Norton MC (2008). Point and 5-year period prevalence of neuropsychiatric symptoms in dementia: the Cache County Study. Int J Geriatr Psychiatry.

[15] Tan LL, Wong HB, Allen H (2005). The impact of neuropsychiatric symptoms of dementia on distress in family and professional caregivers in Singapore. Int Psychogeriatr.

[16] Tatsumi H, Nakaaki S, Torii K, Shinagawa Y, Watanabe N, Murata Y (2009). Neuropsychiatric symptoms predict change in quality of life of Alzheimer disease patients: a two-year follow-up study. Psychiatry Clin Neurosci.

[17] Cummings JL (1997). The Neuropsychiatric Inventory: assessing psychopathology in dementia patients. Neurology.

[18] Cummings JL, Mega M, Gray K, Rosenberg-Thompson S, Carusi DA, Gornbein J (1994). The Neuropsychiatric Inventory: comprehensive assessment of psychopathology in dementia. Neurology.

[19] de Medeiros K, Robert P, Gauthier S, Stella F, Politis A, Leoutsakos J (2010). The Neuropsychiatric Inventory-Clinician rating scale (NPI-C): reliability and validity of a revised assessment of neuropsychiatric symptoms in dementia. Int Psychogeriatr.

[20] Wood S, Cummings JL, Hsu MA, Barclay T, Wheatley MV, Yarema KT (2000). The use of the neuropsychiatric inventory in nursing home residents: Characterization and measurement. Am J Geriatr Psychiatry.

[21] Kaufer DI, Cummings JL, Ketchel P, Smith V, MacMillan A, Shelley T (2000). Validation of the NPI-Q, a brief clinical form of the Neuropsychiatric Inventory. J Neuropsychiatry Clin Neurosci.

[22] Trzepacz PT, Saykin A, Yu P, Bhamditipati P, Sun J, Dennehy EB (2013). Subscale validation of the neuropsychiatric inventory questionnaire: comparison of Alzheimer’s disease neuroimaging initiative and national Alzheimer’s coordinating center cohorts. Am J Geriatr Psychiatry.

[23] Lyketsos CG, Steinberg M, Tschanz JT, Norton MC, Steffens DC, Breitner JC (2000). Mental and behavioral disturbances in dementia: findings from the Cache County Study on Memory in Aging. Am J Psychiatry.

[24] Canevelli M, Adali N, Voisin T, Soto ME, Bruno G, Cesari M (2013). Behavioral and psychological subsyndromes in Alzheimer’s disease using the Neuropsychiatric Inventory. Int J Geriatr Psychiatry.

[25] Vilalta-Franch J, Lopez-Pousa S, Turon-Estrada A, Lozano-Gallego M, Hernandez-Ferrandiz M, Pericot-Nierga I (2010). Syndromic association of behavioral and psychological symptoms of dementia in Alzheimer disease and patient classification. Am J Geriatr Psychiatry.

[26] Aalten P, de Vugt ME, Lousberg R, Korten E, Jaspers N, Senden B (2003). Behavioral problems in dementia: a factor analysis of the neuropsychiatric inventory. Dement Geriatr Cogn Disord.

[27] Aalten P, Verhey FR, Boziki M, Bullock R, Byrne EJ, Camus V (2007). Neuropsychiatric syndromes in dementia. Results from the European Alzheimer Disease Consortium: part I. Dement Geriatr Cogn Disord.

[28] Garre-Olmo J, Lopez-Pousa S, Vilalta-Franch J, de Gracia BM, Vilarrasa AB (2010). Grouping and trajectories of the neuropsychiatric symptoms in patients with Alzheimer’s disease, part I: symptom clusters. J Alzheimers Dis.

[29] Hollingworth P, Hamshere ML, Moskvina V, Dowzell K, Moore PJ, Foy C (2006). Four components describe behavioral symptoms in 1,120 individuals with late-onset Alzheimer’s disease. J Am Geriatr Soc.

[30] Kang HS, Ahn IS, Kim JH, Kim DK (2010). Neuropsychiatric symptoms in Korean patients with Alzheimer’s disease: exploratory factor analysis and confirmatory factor analysis of the neuropsychiatric inventory. Dement Geriatr Cogn Disord.

[31] Spalletta G, Musicco M, Padovani A, Rozzini L, Perri R, Fadda L (2010). Neuropsychiatric symptoms and syndromes in a large cohort of newly diagnosed, untreated patients with Alzheimer disease. Am J Geriatr Psychiatry.

[32] Cheng ST, Kwok T, Lam LC (2012). Neuropsychiatric symptom clusters of Alzheimer’s disease in Hong Kong Chinese: prevalence and confirmatory factor analysis of the Neuropsychiatric Inventory. Int Psychogeriatr.

[33] Insel T, Cuthbert B, Garvey M, Heinssen R, Pine DS, Quinn K (2010). Research domain criteria (RDoC): toward a new classification framework for research on mental disorders. Am J Psychiatry.

[34] Catani M, Ffytche DH (2005). The rises and falls of disconnection syndromes. Brain.

[35] Geda YE, Schneider LS, Gitlin LN, Miller DS, Smith GS, Bell J (2013). Neuropsychiatric symptoms in Alzheimer’s disease: past progress and anticipation of the future. Alzheimers Dement.

[36] Francis PT, Sims NR, Procter AW, Bowen DM (1993). Cortical pyramidal neurone loss may cause glutamatergic hypoactivity and cognitive impairment in Alzheimer’s disease: investigative and therapeutic perspectives. J Neurochem.

[37] Nagahara AH, Merrill DA, Coppola G, Tsukada S, Schroeder BE, Shaked GM (2009). Neuroprotective effects of brain-derived neurotrophic factor in rodent and primate models of Alzheimer’s disease. Nat Med.

[38] Wuwongse S, Chang RC, Law AC (2010). The putative neurodegenerative links between depression and Alzheimer’s disease. Prog Neurobiol.

[39] Lai MK, Tsang SW, Esiri MM, Francis PT, Wong PT, Chen CP (2011). Differential involvement of hippocampal serotonin1A receptors and re-uptake sites in non-cognitive behaviors of Alzheimer’s disease. Psychopharmacology (Berl).

[40] Weinshenker D (2008). Functional consequences of locus coeruleus degeneration in Alzheimer’s disease. Curr Alzheimer Res.

[41] Lyness SA, Zarow C, Chui HC (2003). Neuron loss in key cholinergic and aminergic nuclei in Alzheimer disease: a meta-analysis. Neurobiol Aging.

[42] Zweig RM, Ross CA, Hedreen JC, Steele C, Cardillo JE, Whitehouse PJ (1989). Neuropathology of aminergic nuclei in Alzheimer’s disease. Prog Clin Biol Res.

[43] Thomas AJ, Hendriksen M, Piggott M, Ferrier IN, Perry E, Ince P (2006). A study of the serotonin transporter in the prefrontal cortex in late-life depression and Alzheimer’s disease with and without depression. Neuropathol Appl Neurobiol.

[44] Rodriguez JJ, Noristani HN, Verkhratsky A (2012). The serotonergic system in ageing and Alzheimer’s disease. Prog Neurobiol.

[45] Marner L, Frokjaer VG, Kalbitzer J, Lehel S, Madsen K, Baare WF (2012). Loss of serotonin 2A receptors exceeds loss of serotonergic projections in early Alzheimer’s disease: a combined [11C]DASB and [18F]altanserin-PET study. Neurobiol Aging.

[46] Minger SL, Esiri MM, McDonald B, Keene J, Carter J, Hope T (2000). Cholinergic deficits contribute to behavioral disturbance in patients with dementia. Neurology.

[47] Zweig RM, Ross CA, Hedreen JC, Steele C, Cardillo JE, Whitehouse PJ (1988). The neuropathology of aminergic nuclei in Alzheimer’s disease. Ann Neurol.

[48] Garcia-Alloza M, Tsang SW, Gil-Bea FJ, Francis PT, Lai MK, Marcos B (2006). Involvement of the GABAergic system in depressive symptoms of Alzheimer’s disease. Neurobiol Aging.

[49] Zahodne LB, Gongvatana A, Cohen RA, Ott BR, Tremont G, Alzheimer’s Disease Neuroimaging Initiative (2013). Are apathy and depression independently associated with longitudinal trajectories of cortical atrophy in mild cognitive impairment?. Am J Geriatr Psychiatry.

[50] Lee DY, Choo IH, Jhoo JH, Kim KW, Youn JC, Lee DS (2006). Frontal dysfunction underlies depressive syndrome in Alzheimer disease: a FDG-PET study. Am J Geriatr Psychiatry.

[51] Sultzer DL, Mahler ME, Mandelkern MA, Cummings JL, Van Gorp WG, Hinkin CH (1995). The relationship between psychiatric symptoms and regional cortical metabolism in Alzheimer’s disease. J Neuropsychiatry Clin Neurosci.

[52] Ouchi Y, Yoshikawa E, Futatsubashi M, Yagi S, Ueki T, Nakamura K (2009). Altered brain serotonin transporter and associated glucose metabolism in Alzheimer disease. J Nucl Med.

[53] Khandai AC, Aizenstein HJ (2013). Recent advances in neuroimaging biomarkers in geriatric psychiatry. Curr Psychiatry Rep.

[54] Hirao K, Smith GS (2014). Positron emission tomography molecular imaging in late-life depression. J Geriatr Psychiatry Neurol.

[55] David R, Koulibaly M, Benoit M, Garcia R, Caci H, Darcourt J (2008). Striatal dopamine transporter levels correlate with apathy in neurodegenerative diseases A SPECT study with partial volume effect correction. Clin Neurol Neurosurg.

[56] Sultzer DL, Melrose R, Campa OR, Achamallah N, Harwood D, Brody A (2010). Cholinergic receptor imaging in Alzheimer’s disease: method and early results, in Annual Meeting of the American Association for Geriatric Psychiatry. Am J Geriatr Psychiatry.

[57] Starkstein SE, Mizrahi R, Capizzano AA, Acion L, Brockman S, Power BD (2009). Neuroimaging correlates of apathy and depression in Alzheimer’s disease. J Neuropsychiatry Clin Neurosci.

[58] Marshall GA, Fairbanks LA, Tekin S, Vinters HV, Cummings JL (2006). Neuropathologic correlates of apathy in Alzheimer’s disease. Dement Geriatr Cogn Disord.

[59] Assal F, Alarcon M, Solomon EC, Masterman D, Geschwind DH, Cummings JL (2004). Association of the serotonin transporter and receptor gene polymorphisms in neuropsychiatric symptoms in Alzheimer disease. Arch Neurol.

[60] Serra L, Cercignani M, Lenzi D, Perri R, Fadda L, Caltagirone C (2010). Grey and white matter changes at different stages of Alzheimer’s disease. J Alzheimers Dis.

[61] Bruen PD, McGeown WJ, Shanks MF, Venneri A (2008). Neuroanatomical correlates of neuropsychiatric symptoms in Alzheimer’s disease. Brain.

[62] Trzepacz PT, Yu P, Bhamidipati PK, Willis B, Forrester T, Tabas L (2013). Frontolimbic atrophy is associated with agitation and aggression in mild cognitive impairment and Alzheimer’s disease. Alzheimers Dement.

[63] Reeves S, Brown R, Howard R, Grasby P (2009). Increased striatal dopamine (D2/D3) receptor availability and delusions in Alzheimer disease. Neurology.

[64] Melrose RJ, Ettenhofer ML, Harwood D, Achamallah N, Campa O, Mandelkern M (2011). Cerebral metabolism, cognition, and functional abilities in Alzheimer disease. J Geriatr Psychiatry Neurol.

[65] Sultzer DL, Leskin LP, Melrose RJ, Harwood DG, Narvaez TA, Ando TK (2014). Neurobiology of delusions, memory, and insight in Alzheimer disease. Am J Geriatr Psychiatry.

[66] Mielke MM, Kozauer NA, Chan KC, George M, Toroney J, Zerrate M (2009). Regionally-specific diffusion tensor imaging in mild cognitive impairment and Alzheimer’s disease. Neuroimage.

[67] Balthazar ML, Pereira FR, Lopes TM, da Silva EL, Coan AC, Campos BM (2014). Neuropsychiatric symptoms in Alzheimer’s disease are related to functional connectivity alterations in the salience network. Hum Brain Mapp.

[68] Menon V, Uddin LQ (2010). Saliency, switching, attention and control: a network model of insula function. Brain Struct Funct.

[69] Murray PS, Kumar S, Demichele-Sweet MA, Sweet RA (2014). Psychosis in Alzheimer’s disease. Biol Psychiatry.

[70] Garcia-Alloza M, Gil-Bea FJ, Diez-Ariza M, Chen CP, Francis PT, Lasheras B (2005). Cholinergic-serotonergic imbalance contributes to cognitive and behavioral symptoms in Alzheimer’s disease. Neuropsychologia.

[71] Marcos B, Garcia-Alloza M, Gil-Bea FJ, Chuang TT, Francis PT, Chen CP (2008). Involvement of an altered 5-HT -{6} receptor function in behavioral symptoms of Alzheimer’s disease. J Alzheimers Dis.

[72] Forstl H, Burns A, Levy R, Cairns N (1994). Neuropathological correlates of psychotic phenomena in confirmed Alzheimer’s disease. Br J Psychiatry.

[73] Banno K, Nakaaki S, Sato J, Torii K, Narumoto J, Miyata J (2014). Neural basis of three dimensions of agitated behaviors in patients with Alzheimer disease. Neuropsychiatr Dis Treat.

[74] Rafii MS, Taylor CS, Kim HT, Desikan RS, Fleisher AS, Katibian D (2014). Neuropsychiatric symptoms and regional neocortical atrophy in mild cognitive impairment and Alzheimer’s disease. Am J Alzheimers Dis Other Demen.

[75] Mega MS, Lee L, Dinov ID, Mishkin F, Toga AW, Cummings JL (2000). Cerebral correlates of psychotic symptoms in Alzheimer’s disease. J Neurol Neurosurg Psychiatry.

[76] Devinsky O, Morrell MJ, Vogt BA (1995). Contributions of anterior cingulate cortex to behaviour. Brain.

[77] Allman JM, Hakeem A, Erwin JM, Nimchinsky E, Hof P (2001). The anterior cingulate cortex: the evolution of an interface between emotion and cognition. Ann N Y Acad Sci.

[78] Sepehry AA, Lee PE, Hsiung GY, Beattie BL, Jacova C (2012). Effect of selective serotonin reuptake inhibitors in Alzheimer’s disease with comorbid depression: a meta-analysis of depression and cognitive outcomes. Drugs Aging.

[79] Mokhber N, Abdollahian E, Soltanifar A, Samadi R, Saghebi A, Haghighi MB (2014). Comparison of sertraline, venlafaxine and desipramine effects on depression, cognition and the daily living activities in Alzheimer patients. Pharmacopsychiatry.

[80] Mori T, Shimada H, Shinotoh H, Hirano S, Eguchi Y, Yamada M (2014). Apathy correlates with prefrontal amyloid beta deposition in Alzheimer’s disease. J Neurol Neurosurg Psychiatry.

[81] Drye LT, Scherer RW, Lanctot KL, Rosenberg PB, Herrmann N, Bachman D (2013). Designing a trial to evaluate potential treatments for apathy in dementia: the apathy in dementia methylphenidate trial (ADMET). Am J Geriatr Psychiatry.

[82] Rosenberg PB, Lanctot KL, Drye LT, Herrmann N, Scherer RW, Bachman DL (2013). Safety and efficacy of methylphenidate for apathy in Alzheimer’s disease: a randomized, placebo-controlled trial. J Clin Psychiatry.

[83] Lanctot KL, Chau SA, Herrmann N, Drye LT, Rosenberg PB, Scherer RW (2014). Effect of methylphenidate on attention in apathetic AD patients in a randomized, placebo-controlled trial. Int Psychogeriatr.

[84] Herrmann N, Rothenburg LS, Black SE, Ryan M, Liu BA, Busto UE (2008). Methylphenidate for the treatment of apathy in Alzheimer disease: prediction of response using dextroamphetamine challenge. J Clin Psychopharmacol.

[85] Padala PR, Burke WJ, Shostrom VK, Bhatia SC, Wengel SP, Potter JF (2010). Methylphenidate for apathy and functional status in dementia of the Alzheimer type. Am J Geriatr Psychiatry.

[86] Waldemar G, Gauthier S, Jones R, Wilkinson D, Cummings J, Lopez O (2011). Effect of donepezil on emergence of apathy in mild to moderate Alzheimer’s disease. Int J Geriatr Psychiatry.

[87] Erkulwater S, Pillai R (1989). Amantadine and the end-stage dementia of Alzheimer’s type. South Med J.

[88] Lanctot KL, Herrmann N, Black SE, Ryan M, Rothenburg LS, Liu BA (2008). Apathy associated with Alzheimer disease: use of dextroamphetamine challenge. Am J Geriatr Psychiatry.

[89] Frakey LL, Salloway S, Buelow M, Malloy P (2012). A randomized, double-blind, placebo-controlled trial of modafinil for the treatment of apathy in individuals with mild-to-moderate Alzheimer’s disease. J Clin Psychiatry.

[90] Vilalta-Franch J, Lopez-Pousa S, Calvo-Perxas L, Garre-Olmo J (2013). Psychosis of Alzheimer disease: prevalence, incidence, persistence, risk factors, and mortality. Am J Geriatr Psychiatry.

[91] Trifirò G, Spina E, Gambassi G (2009). Use of antipsychotics in elderly patients with dementia: do atypical and conventional agents have a similar safety profile?. Pharmacol Res.

[92] Schneider LS, Dagerman KS, Insel P (2005). Risk of death with atypical antipsychotic drug treatment for dementia: meta-analysis of randomized placebo-controlled trials. JAMA.

[93] Sultzer DL, Davis SM, Tariot PN, Dagerman KS, Lebowitz BD, Lyketsos CG (2008). Clinical symptom responses to atypical antipsychotic medications in Alzheimer’s disease: phase 1 outcomes from the CATIE-AD effectiveness trial. Am J Psychiatry.

[94] Schneider LS, Tariot PN, Dagerman KS, Davis SM, Hsiao JK, Ismail MS (2006). Effectiveness of atypical antipsychotic drugs in patients with Alzheimer’s disease. N Engl J Med.

[95] Lopez OL, Becker JT, Chang YF, Sweet RA, Aizenstein H, Snitz B (2013). The long-term effects of conventional and atypical antipsychotics in patients with probable Alzheimer’s disease. Am J Psychiatry.

[96] Tsoi T, Baillon S, Lindesay J (2008). Early frontal executive impairment as a predictor of subsequent behavior disturbance in dementia. Am J Geriatr Psychiatry.

[97] Lyketsos CG, Rosenblatt A, Rabins P (2004). Forgotten frontal lobe syndrome or ‘Executive Dysfunction Syndrome’. Psychosomatics.

[98] Hopkins MW, Libon DJ (2005). Neuropsychological functioning of dementia patients with psychosis. Arch Clin Neuropsychol.

[99] Drye LT, Ismail Z, Porsteinsson AP, Rosenberg PB, Weintraub D, Marano C (2012). Citalopram for agitation in Alzheimer’s disease: design and methods. Alzheimers Dement.

[100] Porsteinsson AP, Drye LT, Pollock BG, Devanand DP, Frangakis C, Ismail Z (2014). Effect of citalopram on agitation in Alzheimer disease: the CitAD randomized clinical trial. JAMA.

[101] Imamura T, Takanashi M, Hattori N, Fujimori M, Yamashita H, Ishii K (1998). Bromocriptine treatment for perseveration in demented patients. Alzheimer Dis Assoc Disord.

[102] Kraus MF, Maki PM (1997). Effect of amantadine hydrochloride on symptoms of frontal lobe dysfunction in brain injury: case studies and review. J Neuropsychiatry Clin Neurosci.

[103] Mizukami K, Hatanaka K, Ishii T, Iwakiri M, Sodeyama N, Tanaka Y (2010). Effects of sodium valproate on behavioral disturbances in elderly outpatients with dementia. Geriatr Gerontol Int.

[104] Konovalov S, Muralee S, Tampi RR (2008). Anticonvulsants for the treatment of behavioral and psychological symptoms of dementia: a literature review. Int Psychogeriatr.

[105] Bidzan L, Grabowski J, Dutczak B, Bidzan M (2012). Impact of treatment with acetylcholinesterase inhibitors, valproic acid and antipsychotics on aggressive behaviour in Alzheimer’s type dementia [in Polish]. Psychiatr Pol.

[106] Livingston G, Johnston K, Katona C, Paton J, Lyketsos CG (2005). Systematic review of psychological approaches to the management of neuropsychiatric symptoms of dementia. Am J Psychiatry.

[107] Moniz-Cook E, Woods RT, Richards K (2001). Functional analysis of challenging behaviour in dementia: the role of superstition. Int J Geriatr Psychiatry.

[108] Olazarán J, Reisberg B, Clare L, Cruz I, Peña-Casanova J, Del Ser T (2010). Nonpharmacological therapies in Alzheimer’s disease: a systematic review of efficacy. Dement Geriatr Cogn Disord.

[109] Taragano FE, Allegri RF, Krupitzki H, Sarasola DR, Serrano CM, Lon L (2009). Mild behavioral impairment and risk of dementia: a prospective cohort study of 358 patients. J Clin Psychiatry.

